# Relevance and Effectiveness of Combined Resistance and Balance Training to Improve Balance and Muscular Fitness in Healthy Youth and Youth Athletes: A Scoping Review

**DOI:** 10.1007/s40279-022-01789-7

**Published:** 2022-11-15

**Authors:** Urs Granacher, David G. Behm

**Affiliations:** 1 Department of Sport and Sport Science, Exercise and Human Movement Science, University of Freiburg, Sandfangweg 4, 79102 Freiburg, Germany; 2 School of Human Kinetics and Recreation, Memorial University of Newfoundland, St. John’s, NL Canada

## Abstract

**Background:**

Balance and resistance training applied as single-mode interventions have proven to enhance measures of balance and/or muscular fitness in youth and youth athletes. Less is known on the effectiveness of combined or sequenced balance and resistance training in youth and youth athletes.

**Objective:**

The objective of this scoping review was to describe the effects of concurrently performed balance and resistance training (i.e., metastable resistance training) and different sequencing schemes of balance and resistance training on measures of balance and/or muscular fitness in healthy youth and youth athletes. We additionally aimed to identify knowledge gaps in the literature.

**Methods:**

According to the principles of a scoping review, we followed a broad research question, identified gaps in the literature, and reported findings on the level of single studies but did not aggregate and meta-analyze outcomes across studies. For this purpose, systematic literature searches were conducted in the electronic databases PubMed (including MEDLINE), Web of Science, and SPORTDiscus from inception until August 2022. We included metastable resistance training and sequenced balance and resistance training studies in healthy youth and youth athletes aged 6–18 years that investigated the training-induced effects on measures of balance (e.g., stork balance test) and muscular fitness (e.g., countermovement jump test) in contrast to active/passive controls. The Physiotherapy Evidence Database (PEDro) scale was used to assess the risk of bias. The Strength of Recommendation Taxonomy (SORT) was applied for the whole scoping review on levels A (high strength of recommendation) to C (low strength of recommendation) and for individual studies on levels 1 (high-quality evidence) to 3 (low-quality evidence).

**Results:**

The strength of recommendation for the scoping review was level B based on inconsistent or limited-quality evidence. Eleven randomized controlled trials were eligible to be included in this scoping review and each study was rated as ‘limited-quality evidence’. A median PEDro score of 6 was computed across the included studies. Four studies examined the effects of metastable resistance training (e.g., plyometric training on unstable surfaces) on measures of balance and/or muscle fitness in youth athletes. The remaining seven studies investigated the impact of sequenced balance and resistance training (e.g., blocked balance training followed by blocked resistance training) on measures of balance and/or muscle fitness in youth and youth athletes. The duration of the intervention programs ranged from 6 to 10 weeks with 2-weekly to 3-weekly exercise sessions. Participants’ age range was 10–18 years (mean 15 years). Ten studies examined young male and female athletes from different sports (i.e., basketball, handball, soccer) and one study physical education students. Metastable resistance training compared with plyometric training performed on stable surfaces showed no extra effect on measures of balance and muscular fitness. Stable plyometric training appears to be even more effective to improve jump performance. Sequenced balance and resistance training in the form of a mesocycle of balance followed by plyometric training seems to be particularly effective to improve measures of balance and muscular fitness in young athletes. This scoping review identified knowledge gaps that may indicate future research avenues: (i) comparative studies should be designed to examine how sex, age, maturity status, and expertise level impact on the adaptive potential following metastable resistance training or sequenced balance and resistance training in youth and youth athletes, (ii) at least one established measure of balance and muscular fitness should always be included in study designs to allow future comparison between studies and to aggregate (meta-analyze) findings across studies and (iii) studies are needed that examine neuromuscular and tendomuscular adaptations following these exercise types as well as dosage effects.

**Conclusions:**

According to the results of this scoping review, balance training should be an essential training component for youth that is incorporated with the resistance training exercises or introduced at least a month before resistance and plyometric training within a periodized training program. More research is needed to examine the moderating roles of age, maturity status, and sex.

**Supplementary Information:**

The online version contains supplementary material available at 10.1007/s40279-022-01789-7.

## Key Points

**Table:** 

Based on limited-quality evidence, metastable resistance training (e.g., plyometric training on unstable surfaces) compared with plyometric training performed on stable surfaces appears not to have an extra effect on measures of balance and muscular fitness in youth athletes.
Based on limited-quality evidence, periodizing a training mesocycle (e.g., 4 weeks) of balance prior to plyometric training accentuates balance and muscular fitness training adaptations in youth and youth athletes.
Future research should particularly elucidate the moderating roles of age, maturity status, sex, and expertise level with metastable resistance training or sequenced balance and resistance training in youth and youth athletes.

## Introduction

There is ample evidence from original research [1], systematic reviews [2], and meta-analyses [3] that physical fitness is associated with markers of performance and health in youth. Muscular fitness is a subset of physical fitness and it has been used as an umbrella term for muscle strength, power, and local muscular endurance [4]. Sufficient levels of muscular fitness are needed in youth for motor performance skill development, athletic, and sport-specific performance [5], with some evidence for improved cognitive functioning [6]. Besides muscular fitness, balance constitutes another important component of physical fitness that has previously been discussed in the context of performance enhancement [7] and injury prevention [8] in youth and youth athletes. For performance enhancement, Granacher and colleagues [7] showed that four weeks of balance training versus regular physical education significantly improved postural sway during one-legged standing, countermovement jump (CMJ) height, and maximal isometric leg extensor force in healthy male and female adolescents.

The World Health Organization recommends youth aged 5–17 years to be physically active 60 min per day at moderate-to-vigorous intensities [9]. According to the World Health Organization, aerobic exercise should be prioritized followed by strengthening exercises. Because of changes in the living environment of youth over the past 30–40 years such as urbanization [10] and mediatization [11] with fewer options or less motivation to be physically active, Faigenbaum and colleagues [12] argued that aerobic, strength, and skill (including balance) exercises should be coequally included in youth physical activity recommendations to promote endurance, muscular fitness, and motor skill acquisition. While the effects of aerobic exercises are well established in youth due to the widespread application of World Health Organization recommendations, less is known on the relevance and effectiveness of muscle strengthening and balance exercises in youth [12]. However, there is evidence that these exercise types should be particularly promoted due to negative secular trends in muscle strength/power (i.e., standing long jump, − 11.2%) and static balance (i.e., flamingo balance test, − 15.3%) in youth aged 11–17 years [13, 14]. In accordance with the principle of training specificity [15], resistance training is well-suited to promote muscular fitness and balance training to improve postural control in youth and youth athletes. In their review article, Faigenbaum and Myer [16] showed that resistance training is a safe, effective, and worthwhile activity for children and adolescents. Gebel and colleagues [17, 18] reported safety and effectiveness of balance training in youth in their meta-analysis and narrative review article. Of note, resistance and balance training can be applied as single-mode interventions [19, 20], in combined form as resistance training performed on unstable surfaces (metastable resistance training) [21] or in a sequenced form with balance training delivered prior to resistance training or vice versa [22, 23]. While metastable resistance training appears to be a time efficient training type [24, 25], its effect could be mitigated because power exercises such as plyometrics (i.e., jump training) conducted on unstable surfaces (e.g., balance pads) may prevent the adequate activation of the stretch shortening cycle needed to induce physiological adaptive processes within the neuromuscular system [26]. In addition, plyometric training on unstable surfaces may negatively impact landing mechanics such as increased knee valgus upon landing [27].

Several original studies have been conducted over recent years that examined the effects of balance and resistance training performed concurrently (metastable resistance training) or in sequenced mode on measures of physical fitness in youth and youth athletes [26, 28]. For instance Hammami and colleagues [23] showed that four weeks of blocked balance training followed by four weeks of blocked plyometric training is more effective than the opposite sequencing scheme (plyometrics prior to balance training) to improve jump performance (i.e., three hop test) and balance (i.e., Y-balance test) in youth soccer athletes. However, it is unresolved whether the inclusion of balance exercises into resistance training, either in the form of metastable resistance training or sequenced balance and resistance training programs, produces larger effects than resistance training alone. To date, no systematic review with a meta-analysis or scoping review is available on the effects of combined balance and resistance training in youth and youth athletes. Accordingly, it is timely to summarize the available study findings on metastable resistance training and sequenced balance and resistance training in youth and youth athletes. Therefore, the aim of this scoping review was to describe the performance-enhancing effects of concurrently executed balance and resistance training (i.e., metastable resistance training) and different periodization schemes of balance and resistance training on measures of static and dynamic balance and/or muscular fitness in healthy youth and youth athletes and to provide recommendations on how to best combine balance and resistance training in youth and youth athletes. An additional objective of this scoping review was to identify knowledge gaps in the literature that may help to design future research to ultimately allow the aggregation of study findings from original research in the form of meta-analyses. Of note, injury preventive aspects related to balance and resistance training with youth were beyond the scope of this review.

## Methods

A typical characteristic of scoping review articles is a broad research question and scope [29]. Here, we summarized findings on the effects of concurrently performed balance and resistance training (i.e., metastable resistance training), sequenced balance, and resistance training using different comparators (e.g., resistance training, sport-specific training) on various outcome variables (i.e., muscular fitness, balance) in trained (e.g., soccer players) or untrained (e.g., physical education students) youth. Accordingly, we decided to perform a scoping review with a systematic literature search and not a meta-analysis. A protocol was created using the Preferred Items for Systematic Reviews and Meta-Analyses extension for Scoping Reviews (PRISMA-ScR) according to Tricco and colleagues [30]. The respective checklist was completed and is available in the Open-Source Framework (https://osf.io/2s4ck/). The scoping review protocol was registered on the Open-Source Framework on 26 August, 2022.

### Literature Search Strategy

Systematic literature searches were conducted in the electronic databases PubMed (including MEDLINE), Web of Science, and SPORTDiscus from inception until August 2022. This scoping review was conducted in accordance with the Preferred Reporting Items for Systematic Reviews and Meta-Analyses (PRISMA) statement [31]. Potentially relevant key terms (and synonyms searched for by the MeSH database) were collected through expert opinion and included in the electronic databases in different combinations using a Boolean search strategy with the operators AND, OR:

*resistance training, weight-bearing exercise, strength training, instability resistance training, instability strength training, metastability resistance training, plyometric training, plyometrics, instability plyometric training, metastability plyometric training, balance training, sensorimotor training, proprioceptive training, perturbation training, youth, children, and adolescent.*

The search syntax was adapted according to the specifics of the respective database and can be found in the Electronic Supplementary Material. We additionally checked retrieved articles for cross references.

### Eligibility Criteria and Study Selection

To be eligible for inclusion, articles had to fulfill the following formal criteria: written in English or German, peer-reviewed original research, full text availability. In addition, we followed a PICOS (population, intervention, comparator, outcome, study design) approach to select studies eligible for inclusion (Table 1). The screening of titles, abstracts, and full texts was realized by two authors (UG, DGB) based on the inclusion and exclusion criteria illustrated in Table 1. More specifically, if titles showed any potential relevance of the article to be included in this scoping review, abstracts were screened and an independent decision was made per rater after perusal of the full text. If the two authors did not reach initial agreement, they discussed the study until they achieved a final consensus.

**Table 1:** Inclusion and exclusion criteria according to PICOS

PICOS category	Inclusion criteria	Exclusion criteria
Population	Healthy youth and youth athletes aged 6–18 years (child aged 6–12 years; adolescent aged 13–18 years)	Adults, patient groups
Intervention	Combined or sequenced resistance training for the lower limbs (e.g., plyometric training) with balance training, metastable resistance training (e.g., plyometric training on unstable ground)	Fewer than six exercise sessions, single-mode balance or resistance training if not used as a comparator for combined or sequenced balance and resistance training; effects of nutritional supplements; core strength training
Comparator	Active or passive control	No control group
Outcomes	Measures of muscular fitness (muscle strength, power, muscular endurance) or static and dynamic balance	No reported measures of muscular fitness or balance
Study design	Controlled trials or randomized controlled trials	Uncontrolled study

### Quality Assessment

The methodological quality of the eleven included studies was assessed using the Physiotherapy Evidence Database (PEDro) scale (https://pedro.org.au/). According to Maher and colleagues, PEDro has good reliability and validity [32]. The maximum score of the PEDro scale is 10 points. Scores < 4 indicate poor methodological quality, scores between 4 and 5 indicate fair quality, scores between 6 and 8 indicate good quality, and scores between 9 and 10 indicate excellent quality [33]. Criterion 1 (eligibility criteria) examines external validity. This item is not used to calculate the PEDro score. Criteria 2–9 examine internal validity of the included studies and whether adequate statistical information is provided to interpret the study findings (criteria 10–11). Two independent researchers (UG and DGB) assessed the quality of the studies with high agreement (Spearman rank correlation coefficient *r* = 0.91), and if any conflict arose, the two authors discussed the respective study until they reached consensus.

### Data Extraction and Synthesis of Results

Microsoft Excel (Microsoft Corporation, Redmond, WA, USA) was used for data extraction. One author (UG) coded each study for first author, publication year, participants’ characteristics, sport discipline and expertise status, experimental design, exercise intervention prescription, adverse events, group-specific pre-post changes for balance and muscular fitness outcomes, and inferential statistics (Table 2). The second author (DGB) checked all entries independently. Discrepancies were discussed by the two authors until they reached consensus. In the column ‘inferential statistics’, main effects of time and group-by-time interactions were reported for each study separately using *p*-values and effect sizes (Cohen’s d). The level of statistical significance was set at *p* < 0.05. The practical significance of a study outcome can be described using the following effect size classification scheme: trivial (effect size < 0.2), small (0.2 ≤ effect size < 0.5), medium (0.5 ≤ effect size < 0.8), and large (effect size ≥ 0.8).

**Table 2:** Study characteristics: experimental design and sample size, exercise prescription, pre-post changes, and statistical outcomes (*p*-values and effect sizes [Cohen’s d])

Study	Participants’ characteristics	Sport discipline, expertise status	Experimental design	Exercise intervention prescription	Adverse events	Group-specific pre-post changes (deltas Δ)	Inferential statistics	Level of evidence^a^
**Metastable resistance training (e.g., metastable plyometric training) in youth athletes**								
Büsch et al. [35]	Healthy elite male youth athletes; age: 16–18 years; biological maturity status: post-PHV	Handball; 9.8 years of systematic handball training	RCT; metastable resistance training (*n* = 9) vs stable plyometric training (*n* = 10)	**Metastable resistance training** 10 weeks of training during the in-season with 2 sessions/week; 60–90 min/session; 100–150 jumps/session; plyometric exercises included single and two-legged SJs, CMJs, DJs on BOSU balls, balance pads, and balance beams **Stable plyometric training** 10 weeks of training during the in-season with 2 sessions/week; 60–90 min/session; 100–150 jumps/session; Plyometric exercises comprised single and two-legged SJs, CMJs, and DJs on stable ground	None	**Metastable resistance training** SJ: Δ 4.7% CMJ: Δ 8.5% DJ: Δ 5.3% **Stable plyometric training** SJ: Δ 11.6% CMJ: Δ 3.6% DJ: Δ 11.2%	Main effects of time for SJ and CMJ (all *p* < 0.004, *d* = 1.64 for CMJ, *d* = 2.04 for SJ; no significant group by time interactions for SJ, CMJ, DJ (*p* > 0.05, *d* = 0.72–1.16)	2
Granacher et al. [26]	Healthy sub-elite male youth athletes; age: 15 years; biological maturity status: N/A	Soccer; 4.0 years of systematic soccer training	RCT; metastable resistance training (*n* = 12) vs stable plyometric training (*n* = 12)	**Metastable resistance training** 8 weeks of training during the pre-season with 2 sessions/week; 90 min/session; plyometric exercises included CMJs, DJs, hurdle CMJs and DJs on stability trainers, balance pads, and balance beams **Stable plyometric training** 8 weeks of training during the pre-season with 2 sessions/week; 90 min/session; plyometric exercises comprised CMJs, DJs, hurdle CMJs, and DJs on stable ground	None	**Metastable resistance training** CMJ: Δ 4.5% DJ: Δ 7.8% CoP: Δ -14.8% **Stable plyometric training** CMJ: Δ 12.9% DJ: Δ 11.1% CoP: Δ -6.7%	Main effects of time for CMJ, DJ, CoP (all *p* < 0.016, * d* = 2.88 for CMJ, * d* = 1.68 for DJ, * d* = 1.12 for CoP); no significant group by time interactions for DJ, CoP; for CMJ significant interactions in favour of stable group (*p* = 0.005, * d* = 1.32)	2
Negra et al. [36]	Healthy sub-elite male youth athletes; age: 12–13 years; biological maturity status: pre-PHV	Soccer; 4.0 years of systematic soccer training	RCT; metastable resistance training (*n* = 16) vs stable plyometric training (*n* = 18)	**Metastable resistance training** 8 weeks of training during the in-season with 2 sessions/week, 80–90 min/session; 50–120 jumps/session; plyometric exercises included SLJs, CMJs on balance pads and balance beams **Stable plyometric training** 8 weeks of training during the in-season with 2 sessions/week; 80–90 min/session; 50–120 jumps/session; plyometric exercises comprised SLJs, CMJs on stable ground	None	**Metastable resistance training** CMJ: Δ 7.0% SLJ: Δ 6.0% SYBT: Δ 12.0% UYBT: Δ 19.0% **Stable plyometric training** CMJ: Δ 13.0% SLJ: Δ 6.0% SYBT: Δ 9.0% UYBT: Δ 10.0%	ANCOVA showed no significant between-group difference at post for CMJ, SLJ, SYBT, UYBT (*p* > 0.05, * d* = 0.08–0.81)	2
Negra et al. [37]	Healthy sub-elite male youth athletes; age: 12–13 years; Biological maturity status: pre-PHV	Soccer; 4.0 years of systematic soccer training	RCT; combined metastable and stable plyometric training (*n* = 16) vs stable plyometric training (*n* = 17)	**Combined metastable and stable plyometric training** 8 weeks of training during the in-season with 2 sessions/week; 80–90 min/session; 50–120 jumps/session; plyometric exercises included CMJs, ankle hops forward on stable ground and balance pads, balance beams, and stability trainers **Stable plyometric training** 8 weeks of training during the in-season with 2 sessions/week; 80–90 min/session; 50–120 jumps/session; plyometric exercises included CMJs, ankle hops forward on stable ground	None	**Combined metastable and stable plyometric training** CMJ: Δ 7.1% SLJ: Δ 5.4% SSBT: Δ 34.0% USBT: Δ 84.0% **Stable plyometric training** CMJ: Δ 8.4% SLJ: Δ 25.30% SSBT: Δ 32.0% USBT: Δ 53.0%	ANCOVA showed no significant between-group difference at post for CMJ, SLJ, SSBT (*p* > 0.05, * d* = 0.20–0.41); significant between-group difference at post for USBT (*p* < 0.01, * d* = 1.49) in favour of combined metastable and stable plyometric training	2
**Combined or sequenced balance and resistance training in youth and youth athletes**								
Chaouachi et al. [38]	Healthy boys; age: 12–15 years; biological maturity status: circa and post-PHV	Physical education students	RCT; combined balance and stable plyometric training (*n* = 14) vs stable plyometric training (*n* = 14) vs active control (*n* = 14)	**Combined balance and stable plyometric training** 8 weeks of training with 3 sessions/week in addition to physical education classes with 2 lessons/week; plyometric exercises included CMJs, LJs, DJs, SLLH, and SLS with a focus on minimal ground contact time (40% of exercise time); balance exercises were combined with plyometric exercises with an emphasis on proper landing for 3 s after the performance of plyometrics (60% of exercise time) **Stable plyometric training** 8 weeks of training with 3 sessions/week in addition to physical education classes with 2 lessons/week; plyometric exercises included CMJs, LJs, DJs, SLLH, and SLS with a focus on minimal ground contact time (100% of exercise time) **Active control** 8 weeks of regular physical education classes; 2 lessons/week	None	**Combined balance and stable plyometric training** CMJ: Δ 14.1% SLJ: Δ 10.8% SEBT: Δ 7.7% SSBT: Δ 87.9% **Stable plyometric training** CMJ: Δ 11.7% SLJ: Δ 9.6% SEBT: Δ 6.3% SSBT: Δ 59.6% **Control** CMJ: Δ 1.7% SLJ: Δ 0.6% SEBT: Δ 2.3% SSBT: Δ 22.3%	For CMJ, SLJ, SEBT, SSBT within-group magnitude-based inferences showed large (combined balance and plyometric training, * d* = 0.88–1.62), moderate (stable plyometric training, * d* = 0.64–0.79) and unclear effects (control, * d* = 0.05–0.36)	2
Hammami et al. [23]	Healthy elite male youth athletes; age: 12–13 years; biological maturity status: pre-PHV	Soccer; national level elite players	RCT; mesocycle of balance followed by mesocycle of stable plyometric training (*n* = 12) vs mesocycle of stable plyometric followed by mesocycle of balance training (*n* = 12)	**Mesocycle balance followed by stable plyometric training** 4 weeks of balance training followed by 4 weeks of plyometric training during the in-season with 2 sessions/week; balance exercises included kneeling on a Swiss ball, single-legged and two-legged stance on unstable surfaces (e.g., BOSU ball); plyometric exercises comprised CMJs, DJs, LJs, and hurdle jumps; 40–75 jumps/plyometric session **Mesocycle stable plyometric followed by balance training** 4 weeks of plyometric training followed by 4 weeks of balance training during the in-season with 2 sessions/week; Plyometric exercises included CMJs, DJs, LJs, hurdle jumps; 40–75 jumps/plyometric session; balance exercises comprised kneeling on a Swiss ball, single and two-legged stance on unstable surfaces (e.g., BOSU ball)	None	**Mesocycle balance-plyometric training** CMJ: Δ 14.3% SLJ: Δ 18.6% SSBT: Δ 169.5% SYBT: Δ 29.5% **Mesocycle plyometric-balance training** CMJ: Δ 8.6% SLJ: Δ 16.8% SSBT: Δ 130.2% SYBT: Δ 22.0%	Main effects of time for CMJ, SLJ, SSBT, SYBT (all *p* < 0.01, * d* = 1.71 for CMJ, * d* = 3.49 for SLJ, * d* = 2.38 for SSBT, * d* = 5.29 for SYBT); significant group by time interactions for THT, SYBT in favor of the balance- plyometric training group (all *p* < 0.05, * d* = 2.15 for THT, * d* = 0.87 for SYBT)	2
Chaouachi et al. [22]	Healthy elite male youth athletes; age: 13–14 years; biological maturity status: circa-PHV	Soccer; national level elite players	RCT; within-session alternated balance and stable plyometric exercises (*n* = 13) vs within-session blocked balance followed by blocked stable plyometric exercises (*n* = 13)	**Alternated balance followed by stable plyometric exercises** 8 weeks of combined balance and plyometric training applied as alternating exercise pairs within training sessions during the in-season with 2 sessions/week; balance exercises included kneeling on a Swiss ball, one-legged stance, leg bridge on the Swiss ball, lunge on BOSU; Plyometric exercises comprised CMJs, DJs, LJs, and lateral hops **Blocked balance followed by stable plyometric exercises** 8 weeks of combined balance and plyometric training applied as blocked balance exercises followed by blocked plyometric exercises within a single exercise session during the in-season with 2 sessions/week; Balance exercises included kneeling on a Swiss ball, one-legged stance, leg bridge on the Swiss ball, lunge on BOSU; Plyometric exercises comprised CMJs, DJs, LJs, and lateral hops	None	**Alternated balance-plyometric exercises** CMJ: Δ 20.0% SLJ: Δ 12.0% SSBT: Δ 159.0% SYBT: Δ 12.0% **Blocked balance-plyometric exercises** CMJ: Δ 25.0% SLJ: Δ 12.0% SSBT: Δ 139.0% SYBT: Δ 6.0%	Main effects of time for CMJ, SLJ, SSBT, SYBT (all *p* < 0.01, * d* = 1.40 for CMJ, * d* = 3.30 for SLJ, * d* = 1.82 for SSBT, * d* = 2.44 for SYBT); significant group by time interaction for SYBT in favour of the alternated balance—plyometric exercise group (*p* = 0.02, * d* = 0.82)	2
Makhlouf et al. [41]	Healthy elite male youth athletes; age: 10–12 years; biological maturity status: pre-PHV	Soccer; national level elite players	RCT; within session blocked balance followed by stable plyometric exercises (*n* = 21) vs within-session blocked agility followed by blocked stable plyometric exercises (*n* = 20) vs active control (*n* = 16)	**Blocked balance followed by stable plyometric exercises** 8 weeks of combined balance and plyometric training applied as blocked balance exercises followed by blocked plyometric exercises within a single exercise session during the in-season with 2 sessions/week; Balance exercises included kneeling on a Swiss ball, one-legged stance, leg bridge on the Swiss ball, lunge on BOSU; Plyometric exercises comprised CMJs, DJs, LJs, and lateral hops **Blocked agility followed by stable plyometric exercises** 8 weeks of combined agility and plyometric training applied as blocked agility exercises followed by blocked plyometric exercises within a single exercise session during the in-season with 2 sessions/week; agility exercises included different ladder drills, with CoD tasks in response to an external stimuli; plyometric exercises comprised CMJs, DJs, LJs, and lateral hops **Active control** 8 weeks of regular soccer training with comparable training volumes as intervention groups; 2 sessions/week	None	**Blocked balance-plyometric exercises** CMJ: Δ 18.4% THT: Δ 9.2% SSBT: Δ 145.0% SYBT: Δ 14.7% **Blocked agility-plyometric exercises** CMJ: Δ 13.4% THT: Δ 8.4% SSBT: Δ 210.5% SYBT: Δ 10.9%	Main effects of time for CMJ, THT, SSBT, SYBT (all *p* < 0.001, * d* = 3.13 for CMJ, * d* = 3.34 for THT, * d* = 1.96 for SSBT, * d* = 2.56 for SYBT); significant group by time interaction for CMJ and SYBT in favour of the blocked balance—plyometric exercise group (*p* < 0.002, * d* = 1.03–1.32); significant group by time interaction for SSBT in favour of the blocked agility—plyometric exercise group (*p* < 0.001, * d* = 1.13)	2
Muehlbauer et al. [28]	Healthy sub-elite male youth athletes; age: 13 years; biological maturity status: circa-PHV	Soccer; 2nd division players	RCT; mesocycle of balance followed by mesocycle of stable plyometric training (*n* = 8) vs microcycle of balance followed by microcycle of stable plyometric training (*n* = 9)	**Mesocycle of balance followed by mesocycle of stable plyometric training** 6 weeks of combined balance and plyometric training applied as a 3-week mesocycle of balance training followed by a 3-week mesocycle of plyometric training during the off-season with 2 sessions/week; balance exercises included the one-legged stance on unstable surfaces, backward beam walk; plyometric exercises comprised ankle jumps, SJs, skater jumps **Microcycle of balance followed by microcycle of stable plyometric training** 6 weeks of combined balance and plyometric training applied in alternated sequence with a microcycle of balance training followed by a microcycle of plyometric training during the off-season with 2 sessions/week; Balance exercises included the one-legged stance on unstable surfaces, backward beam walk; plyometric exercises comprised ankle jumps, SJs, skater jumps	Two players reported competition-related injuries	**Mesocycle balance-plyometric training** SJ: Δ 9.0% CMJ: Δ 10.2% DJ: Δ 22.7% SYBT: Δ 5.0% **Microcycle balance-plyometric training** SJ: Δ 1.2% CMJ: Δ 7.3% DJ: Δ 9.3% SYBT: Δ 4.9%	Main effects of time for SJ, CMJ, DJ, SYBT (all *p* < 0.03, * d* = 1.36 for SJ, * d* = 2.21 for CMJ, * d* = 1.96 for DJ, * d* = 1.38 for SYBT); no significant group by time interactions for SJ, CMJ, DJ, SYBT (all *p* > 0.05, * d* = 0.99 for SJ, * d* = 0.32 for CMJ, * d* = 0.74 for DJ, * d* = 0.06 for SYBT)	2
Bouteraa et al. [39]	Healthy sub-elite female youth athletes; age: 16 years; biological maturity status: post-PHV	Basketball; regional level sub-elite players	RCT; combined balance and stable plyometric training (*n* = 16) vs regular basketball training (*n* = 10)	**Combined balance and stable plyometric training** 8 weeks of combined balance and stable plyometric training with 3 balance (i.e., kneeling on a Swiss ball, one-legged stance, chest pass balance exercise) followed by 3 plyometric exercises (i.e., vertical jump and reach, double leg zig zag jump, DJs) during the in-season with 2 sessions/week in addition to regular training **Regular basketball training** 8 weeks of standard basketball training with similar training volume compared with the experimental group	None	**Combined balance-plyometric training** SJ: Δ 10.3% CMJ: Δ 7.3% DJ: Δ 15.2% SSBT: Δ 127.2% SYBT: Δ 9.7% **Basketball training** SJ: Δ − 1.8% CMJ: Δ − 3.5% DJ: Δ − 0.7% SSBT: Δ 11.0% SYBT: Δ 1.7%	Main effects of time for DJ, SSBT, SYBT (all *p* < 0.05, * d* = 0.096 for DJ, * d* = 0.96 for SSBT, * d* = 0.123 for SYBT); significant group by time interactions for DJ (*p* = 0.016, * d* = 0.115) and SSBT (*p* = 0.01, * d* = 0.77) in favor of the combined balance and plyometric training	2
Chaabene et al. [40]	Healthy elite female youth athletes; age: 17 years; biological maturity status: post-PHV	Handball; national level elite players; 8.0 years of systematic handball training	RCT; balance and complex training (*n* = 11) vs complex training (*n* = 12)	**Combined balance and complex training** 8 weeks of combined balance and complex training during the in-season with 2 sessions/week; Balance exercises included single leg balance stance on unstable surfaces directly followed by complex training exercises in the form of back half squats at 80% 1-RM directly followed by 3–4 sets with 6–10 reps of CMJs **Complex training** 8 weeks of complex training during the in-season with 2 sessions/week; complex training included back half squats at 80% 1-RM directly followed by 3–4 sets with 6–10 reps of CMJs	None	**Combined balance and complex training** CMJ: Δ 3.2% SLJ: Δ 6.2% SYBT: Δ 1.0% **Complex training** CMJ: Δ 7.1% SLJ: Δ 6.2% SYBT: Δ − 0.5%	Main effects of time for CMJ, SLJ (all *p* < 0.002, * d* = 1.50 for CMJ, * d* = 1.70 for SLJ); significant group by time interaction for SYBT (*p* = 0.007, * d* = 1.30) in favor of the combined balance and complex training; no significant group by time interactions for CMJ, SLJ (*p* > 0.05, * d* = 0.60 for CMJ, * d* = 0.40 for SLJ)	2

*1-RM* one-repetition maximum, *ANCOVA* analysis of covariance, *CMJ* countermovement jump, *CoD* change of direction, *CoP* center of pressure displacements during balancing, *DJ* drop jump, *LJ* line jump, *N/A* not applicable, *OLDJ* one leg distance jump, *PEDro* Physiotherapy Evidence Database, *PHV* peak height velocity, *RCT* randomized controlled trial, *reps* repetitions, *SEBT* star excursion balance test, *SJ* squat jump, *SLCJ* single-leg cone jumps, *SLJ* standing long jump, *SLLH* single-legged line hop, *SLS* single-legged squats, *SSBT* stork balance test on stable ground, *SYBT* Y balance test on stable ground, *THT* triple hop test, *USBT* stork balance test on unstable ground, *UYBT* Y balance test on unstable ground

^a^Level of evidence for individual studies was rated according to the Strength of Recommendation Taxonomy (SORT) [34]. Level 1 is indicative of good-quality evidence, which is present with high-quality RCTs (i.e., allocation concealed, blinding, intention-to-treat analysis, adequate statistical power, adequate follow-up). See PEDro scores in Table 3. Level 2 means limited-quality evidence which is present with lower quality clinical trials, cohort studies, or case–control studies. Level 3 stands for other evidence (i.e., consensus guidelines, usual practice, opinion)

As an evidence rating system, the Strength of Recommendation Taxonomy (SORT) developed by Ebell and colleagues [34] was used. With SORT, authors can rate the quality, quantity, and consistency of evidence and SORT allows the rating of individual studies and bodies of evidence (e.g., scoping reviews). Given that we aimed to present both, the evidence level of individual studies and the overall strength of recommendation for this scoping review, SORT appears appropriate. The overall strength of recommendation for the scoping review has three levels. SORT A-level recommendation is based on consistent and good-quality evidence; SORT B-level recommendation is based on inconsistent or limited-quality evidence; and SORT C-level recommendation is based on consensus, usual practice, opinion, or case series for studies of diagnosis, treatment, prevention, or screening [34]. The evidence level of individual studies was rated on three levels as well. SORT level 1 stands for good-quality evidence indicated for instance through systematic reviews and meta-analysis or high-quality randomized controlled trials (RCTs) with consistent findings adhering to concealed allocation, blinding, intention-to-treat analysis, adequate statistical power, and adequate follow-up. SORT level 2 denotes limited-quality evidence indicated for instance through lower quality clinical trials, cohort studies, or case–control studies. SORT level 3 is indicative of other evidence, which includes consensus guidelines, usual practice, opinion, and case series. In this scoping review, we rated the level of evidence for an individual study and the strength of recommendation for the whole body of evidence (i.e., scoping review) according to the algorithms provided by Ebell et al. [34]. Certainty of evidence was not rated because we did not compute a meta-analysis.

## Results

### Study Characteristics and Strength of Recommendation

**Fig. 1:**
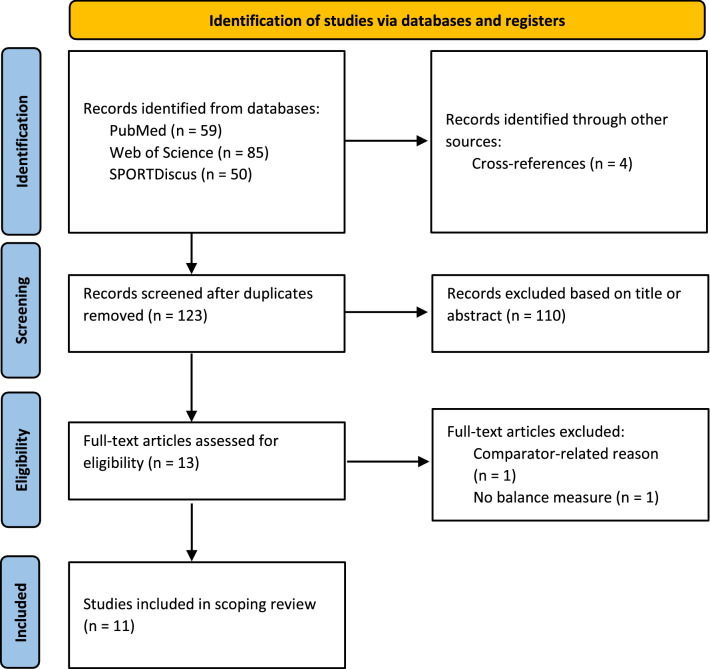
Preferred reporting items for systematic reviews and meta-analyses (PRISMA) flow diagram illustrating the search process

The computerized search in the three electronic databases identified 194 hits with 59 studies detected in PubMed, 85 in Web of Science, and 50 in SPORTDiscus. The literature searches were conducted within each database. The hits per database were imported in the reference management software CITAVI (Copyright © 2022 by Swiss Academic Software GmbH). Seventy-five duplicates were eliminated in CITAVI using the find duplicates function so that 119 potentially eligible hits remained. Cross-referencing of already identified studies revealed another four eligible studies [26, 35–37]. Accordingly, 123 studies were screened for titles, abstracts, and full texts. Finally, eleven studies (all RCTs) were included in this scoping review (Table 2). Figure 1 illustrates a PRISMA flow chart with information on the specifics of the search process. All included RCTs showed limited-quality evidence (level 2) owing to a lack of concealed allocation, blinding, or intention-to-treat analysis (Tables 2 and 3). The strength of recommendation for the whole scoping review achieved SORT level B because of inconsistent or limited-quality evidence. The median PEDro score was 6 across the eleven studies, which is indicative of good overall quality (Table 3). The lowest score was 5, which is indicative of fair quality [38, 39] and the highest score was 8, which denotes good quality [40].

**Table 3:** Physiotherapy evidence database (PEDro) score of the included intervention studies

Study	Eligibility criteria	Random allocation	Concealed allocation	Baseline comparability	Blind subjects	Blinded therapists	Blind assessors	Adequate follow-up	Intention-to-treat	Between-group comparisons	Point estimates and variability	PEDro sum
**Metastable resistance training (e.g., metastable plyometric training) in youth athletes**												
Büsch et al. [35]	●	●	○	●	○	○	○	●	●	●	●	6
Granacher et al. [26]	●	●	○	●	○	○	○	●	●	●	●	6
Negra et al. [36]	●	●	○	●	○	○	○	●	●	●	●	6
Negra et al. [37]	●	●	○	●	○	○	○	●	●	●	●	6
**Combined or sequenced balance and resistance training in youth and youth athletes**												
Chaouachi et al. [38]	●	●	○	●	○	○	○	●	○	●	●	5
Hammami et al. [23]	●	●	○	●	○	○	○	●	●	●	●	6
Chaouachi et al. [22]	●	●	○	●	○	○	○	●	●	●	●	6
Makhlouf et al. [41]	●	●	○	●	○	○	○	●	●	●	●	6
Muehlbauer et al. [28]	●	●	○	●	○	○	○	●	●	●	●	6
Bouteraa et al. [39]	●	●	○	●	○	○	○	●	○	●	●	5
Chaabene et al. [40]	●	●	○	●	●	○	●	●	●	●	●	8

● Adds a point to the PEDro score, ○ adds no point to the score. The item “eligibility criteria” is per definition not included in the final score

A total of 202 participants with an age range of 10–18 years and a mean age of 15 years completed metastable resistance training or sequenced balance and resistance training. The duration of the intervention programs ranged from six to ten weeks and lasted on average eight weeks with two to three sessions per week (mean 2.1 sessions/week). Seven studies were conducted with male sub-elite [26, 28, 36, 37] or elite [22, 23, 41] youth soccer players. Two studies were undertaken with male [35] or female [40] elite handball players, one study with female sub-elite basketball athletes [39], and one study with male physical-education students [38]. Four studies examined pre-pubertals [23, 36, 37, 41], three studies post-pubertals [35, 39, 40], and another three studies circa to post-pubertals [22, 28, 38]. No training or test-related injuries were reported in all included studies. Only one study registered competition-related injuries in two players [28].

Four studies examined the effects of metastable resistance training (e.g., plyometric training on unstable surfaces) on measures of balance and/or muscle strength in youth athletes [26, 35–37]. The remaining seven studies investigated the impact of sequenced balance and resistance training (e.g., blocked balance training followed by blocked resistance training) on measures of balance and/or strength in the general youth population and in youth athletes [22, 23, 28, 38–41]. Only active control groups were used to contrast training-induced gains in metastable resistance training or sequenced balance and resistance training with either single-mode resistance training on a stable surface (e.g., plyometric training, complex training) [26, 35–38, 40] or different sequencing schemes of balance and resistance training [22, 23, 28, 41] or regular sport-specific training [39] or physical education [38].

### Metastable Resistance Training in Youth Athletes

**Fig. 2:**
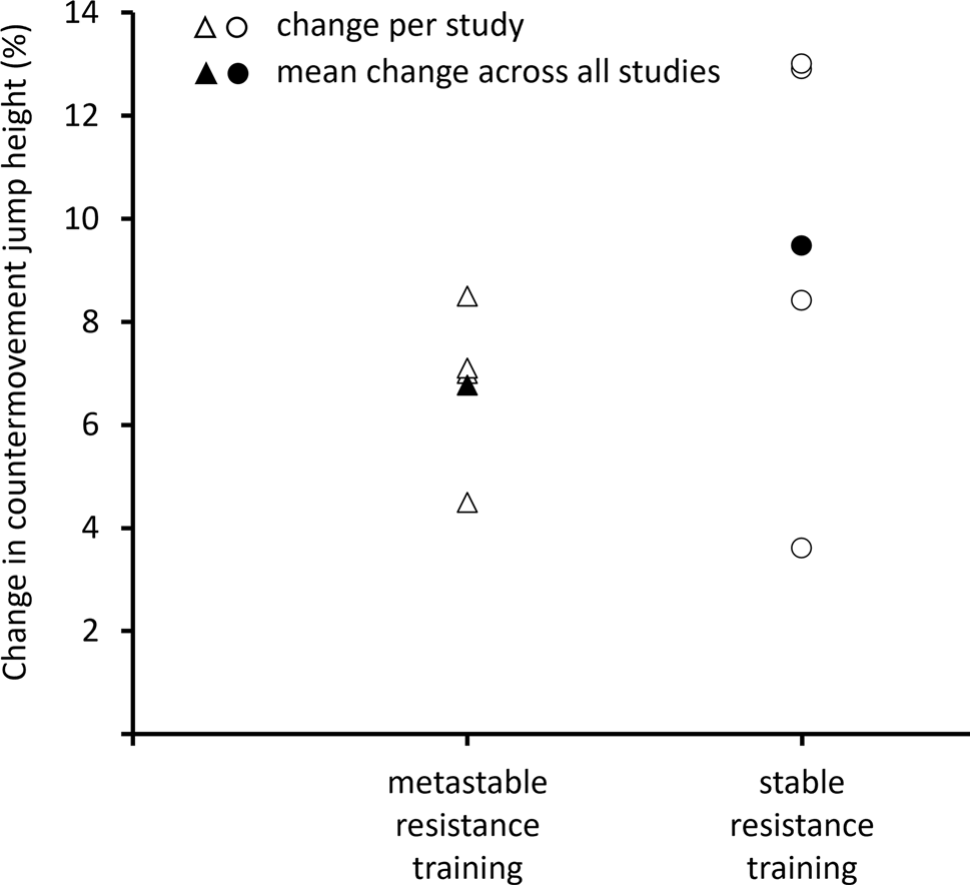
Unweighted percentage change in countermovement jump following metastable (*n* = 4) or stable resistance training (*n* = 4)

Table 2 summarizes four studies showing the effects of metastable or combined metastable and stable resistance training versus single-mode resistance training on stable surfaces on measures of balance and/or muscle strength in youth athletes. The identified metastable resistance training studies were conducted in male youth athletes only and comprised pre-pubertal [36, 37] or post-pubertal [35] soccer [26, 36, 37] or handball players [35] with a total of 53 youth athletes. Overall, the four included studies showed similar improvements for metastable versus stable resistance training for most measures of balance and muscular fitness (Table 2). One study found significantly larger improvements following plyometric training on stable surfaces for CMJ height in post-pubertal handball players [35]. Mean and unweighted changes in CMJ height across all studies showed larger percent changes following stable resistance training (9.5%) versus metastable resistance training (6.8%) [Fig. 2]. In terms of balance, Negra and colleagues [37] observed significant between-group differences at post-test for the stork balance test on an unstable surface in favor of the combined metastable and stable plyometric training group in prepubertal male soccer players (Table 2).

### Sequenced Balance and Resistance Training in Youth and Youth Athletes

Table 2 illustrates seven studies that examined the effects of sequenced balance and resistance training on measures of balance and/or muscle fitness in healthy youth and youth athletes. Two out of seven studies were conducted in pre-pubertal soccer players [23, 41], two studies in circa-pubertal soccer athletes [22, 28], one study in circa and post-pubertal physical-education students [38], and two studies in post-pubertal basketball [39] and handball players [40] with a total of 149 youth practicing combined or sequenced balance and resistance training. Three studies compared the effects of sequenced balance and resistance training with single-mode plyometric training on stable surfaces [38], sport-specific training [39], and single-mode complex training [40]. The combination of balance and resistance training resulted in larger performance improvements for measures of muscle fitness and balance compared with single-mode resistance and sport-specific training in circa and post-pubertal youth (Table 2). The four remaining studies specifically examined sequencing effects of combined balance and resistance training in youth. While three studies showed significant effects in favor of a specific sequencing scheme [22, 23, 41], one study did not [28]. Hammami and colleagues [23] investigated the impact of four weeks of balance followed by four weeks of plyometric training versus four weeks of plyometrics followed by four weeks of balance training. Significantly larger gains were found for triple hop test and Y balance test performance for the balance-plyometric sequencing group (Table 2). Chaouachi et al. [22] examined the effects of within-session sequencing of balance and plyometric exercises either in an alternated or blocked form for eight weeks. The group that performed paired balance and plyometric exercises within an exercise session (alternated sequence) showed larger performance improvements in Y-balance test performance compared with the group that exercised balance first followed by plyometrics (blocked sequence). Finally, Makhlouf and colleagues [41] contrasted blocked balance and plyometric training within training sessions with blocked agility and plyometric training for eight weeks. While the balance-plyometric group achieved larger CMJ height and Y-balance test improvements, the agility-plyometric group showed larger improvements in stork balance test performance (Table 2).

### Knowledge Gaps in the Literature

Table 4 illustrates the six identified knowledge gaps on the effects of metastable resistance training or sequenced balance and resistance training in youth and youth athletes and the respective implications for future research.

**Table 4:** Identified knowledge gaps in the literature and recommendations for future studies

Identified knowledge gaps from scoping review	Implications for future research
Lack of comparative studies that examined MRT or SBRT effects according to age and maturity status	Comparative studies are needed that contrast MRT or SBRT effects according to chronological age and/or maturity status (pre pubertals vs circa vs post-pubertals)
Lack of comparative studies that examined MRT or SBRT effects according to sex	Comparative studies are needed that contrast MRT or SBRT effects according to sex (female vs male)
Lack of comparative studies that examined MRT or SBRT effects according to expertise status	Comparative studies are needed that contrast MRT or SBRT effects according to expertise status (trained vs untrained)
Lack of studies that examined physiological adaptive processes following MRT or SBRT in youth and youth athletes	Elucidate neuromuscular and tendomuscular mechanisms following MRT or SBRT in youth and youth athletes according to sex and maturity status
Inconsistent reporting of balance and muscular fitness outcomes	Always include at least one established measure of balance (e.g., timed single leg stance test, Y-balance test) and muscular fitness (e.g., countermovement jump test, standing long jump test) to allow comparison between studies and to aggregate (meta-analyze) findings across studies in future research
Lack of comparative studies and/or meta-analyses that examined MRT or SBRT dosage effects	Comparative studies and/or future meta-analyses are needed that elucidate the dosage effects of MRT or SBRT

*MRT* metastable resistance training (resistance training on unstable surfaces), *SBRT* sequenced balance and resistance training (e.g., blocked balance training followed by blocked resistance training)

## Discussion

This scoping review examined the effects of balance and resistance training either performed concurrently (i.e., metastable resistance training) or in combination using different sequencing schemes on measures of balance and/or muscular fitness in healthy youth or youth athletes. Based on limited-quality evidence, it is suggested that metastable resistance training compared with plyometric training performed on stable surfaces appears not to have an extra effect on measures of balance and muscular fitness. Preliminary evidence from one study indicates that plyometric training on stable surfaces seems to be even more effective to improve jump performance. Moreover, a mesocycle of balance followed by plyometric training appears to be particularly effective to improve measures of balance and muscular fitness in youth. The SORT level of evidence indicated good-quality evidence for all individual studies (Table 2). The strength of recommendation for the whole scoping review is inconsistent with limited-quality evidence. Consequently, comparative studies are needed in future research that contrast the metastable resistance training or sequenced balance and resistance training effects according to age, maturity status, sex, and expertise status.

### Metastable Resistance Training in Youth Athletes

Of the four studies that examined the effects of metastable resistance training on measures of balance and/or muscular fitness, all were conducted in youth athletes and none in the general youth population. This can most likely be explained by the fact that the performance of plyometrics on unstable surfaces is rather challenging and only trained individuals appear to be ready to perform such demanding exercise drills. Accordingly, the following discussion primarily focusses on youth athletes and not on the general youth population.

A major finding of this scoping review is that metastable resistance training had no extra positive effects on measures of balance and muscular fitness compared with plyometric training on stable surfaces in male youth athletes. Given that only male youth athletes were examined and study outcomes were similar irrespective of the maturity status under investigation, future comparative studies are needed to elucidate the moderating role of sex, maturity status, and expertise level (Table 4).

The similar effects of metastable resistance training and plyometric training on stable surfaces can likely be attributed to the degree of metastability in each of the two activities. Metastability has been defined as the state in which a system remains for a prolonged period (stable instability), and any slight disturbance that may cause the system to deviate from the metastable state does not result in the system passing into another state (unstable) [42]. As soon as the external disturbance is removed, the system will return to the initial metastable state. Kibele et al. [21] further explained that metastability is a state of flux, which allows the transfer from relatively stable to relatively unstable conditions with a return to a more stable condition (e.g., change from stance to flight phase during running). Whilst plyometric training in the extracted studies for this review was performed on stable surfaces and contrasted with metastable resistance training, it does not necessarily connote that the activities were actually fully stable. Plyometric activities such as drop jumps, CMJ, hurdle jumps, hopping, bounding, and other similar activities necessitate a dynamic movement of the center of gravity from its initial stable position (over the base of support) to positions that can be above, below, lateral, in front, or behind the original position including outside the personal base of support. Hence, performing plyometric activities on a stable surface can still be considered a metastable activity, which can stress and induce balance-related training adaptations.

The degree of metastability with plyometrics will depend on the proficiency of the athlete. Typically, youth do not have fully matured balance control systems [20, 43] and thus would not have the extent of plyometric expertise and metastable control compared with a highly trained adult with more training years. Thus, youth would experience plyometric training as a greater metastable training stimulus than a similarly trained adult and thus a suitable activity for improving dynamic balance with similar effects to metastable resistance training. Consequently, metastable resistance training has been applied only in male youth athletes but not in the general youth population.

In support of this contention, although Almeida et al. [44] did not specifically measure balance, they reported that twelve weeks (twice per week for 20 min per day) of plyometric training improved components of gross motor coordination in children aged 7–9 years. Hammami et al. [45] had handball players perform plyometric training either on a stable or sand surface for seven weeks. Although the plyometric training on sand group showed some greater balance training benefits, both plyometric groups improved dynamic and static balance. Another seven-week (three times per week) resistance training program demonstrated similar positive resistance training effects (1 repetition maximum strength, power, movement velocity and jumping ability) when comparing training on stable versus unstable surfaces in healthy male adults [46]. Similarly, eight weeks of plyometric training either on a stable surface (floor) or unstable surfaces (variety of wobble boards, inflatable discs, and others) induced similar improvements in balance, speed, and agility in sub-elite adolescent soccer players [26]. After inducing fatigue with double-leg box jumps to failure, fatigue-induced changes in drop jump and CMJ performance were similar whether performed on stable or unstable surfaces in jump-trained elite volleyball athletes [47]. Hence, physically demanding training activities involving high load or intensity, closed kinetic chain exercises such as plyometrics provide similar training benefits as metastable resistance training as many of these closed kinetic chain exercises on stable surfaces provide substantial metastable challenges. These conclusions are in accord with a meta-analysis [48] reporting that plyometric jump training provided small positive effects on overall balance and dynamic and static balance. These beneficial plyometric training effects on balance were similar to other exercise types (i.e., balance training), irrespective of sex and participants’ age.

The maintenance of balance entails both upstream afferent information to the vestibular system and the downstream efferent system to activate and respond with muscular contractions to impose movement corrections to maintain the metastable state [49, 50]. While there is substantial evidence that performing exercise on unstable surfaces can induce substantial core (trunk) and limb joint muscle activation [24, 51], there is also evidence that high-intensity closed kinetic chain activities such as squats, deadlifts, and plyometrics can also elicit high core (trunk) muscle and limb electromyography (EMG) activity. Hamlyn et al. [52] compared squats and deadlifts using 80% of the 1 repetition maximum to a variety of calisthenic exercises performed on unstable Swiss (exercise) balls and found greater erector spinae EMG activity with the squats and deadlifts performed on stable surfaces than the “unstable” calisthenic exercises. There were no significant muscle activation differences with the gluteus medius, tibialis anterior, and peroneus longus regardless of whether young adults dropped from a 30-cm platform onto a stable or unstable surface [53, 54]. In contrast to reports of increased EMG activity with unstable exercises, greater surface instability produced decreased calf (e.g., tibialis anterior, gastrocnemius, and soleus) and thigh (e.g., vastus medialis, vastus lateralis, and biceps femoris) EMG activity during pre-activation and short latency response periods (30–60 ms post-landing) with drop jump landings from 20-cm, 40-cm, or 60-cm platforms [54], and during braking and push-off phases from 40-cm drop jumps [27] when landing on unstable compared to stable surfaces. While lower intensity metastable exercises have been shown to induce higher muscle activation than similar exercises performed on stable surfaces [25, 51], the aforementioned EMG studies again emphasize the similarity of muscular responses to plyometric activities performed on stable versus unstable surfaces.

Evidence from one study indicates that plyometric training on a stable surface is even more effective to improve jump performance [35], which is likely related to the training specificity principle. Greater CMJ height is highly dependent upon an effective stretch–shortening cycle [55]. In order to take advantage of the stretch reflex and the elastic rebound of the muscle and connective tissue [56, 57], the amortization or transition period from eccentric to concentric contractions must be of a short duration [58]. The performance of drop jumps onto unstable or more compliant surfaces has often been reported to increase the duration of the braking phase (eccentric component of landing) [27, 47, 54], which has also been measured and noted as decreased knee flexion velocities [54]. Moritz and Farley [59] observed hopping on a variety of soft elastic surfaces and reported reduced knee extensor muscles pre-stretch adversely affecting the stretch–shortening cycle. Hence, the advantage of plyometric exercises on stable surfaces is a relatively metastable activity performed with brief amortization periods that incorporate an effective stretch–shortening cycle.

### Combined or Sequenced Balance and Resistance Training in Youth and Youth Athletes

Seven studies examined combined or sequenced balance and resistance training effects in youth and youth athletes [22, 23, 28, 38–41]. Five studies were conducted with boys only [22, 23, 28, 38, 41] and two studies with girls [39, 40]. Two studies were performed with pre-pubertal boys [23, 41], three studies with circa-pubertal boys [22, 28, 38], and another two studies with post-pubertal girls [39, 40]. One study was conducted with physical education students [38] and six studies with youth athletes [22, 23, 28, 39–41]. Because of the large heterogeneity of the included studies, it was impossible to elucidate the effects of maturity status, sex, and expertise level. Future comparative studies are needed that contrast for instance male versus female individuals or trained versus untrained youth or pre-pubertals versus circa-pubertals (Table 4).

The present review suggests that a mesocycle of balance followed by plyometric training appears to be particularly effective to improve measures of balance and muscular fitness in youth and youth athletes. As youth’s balance and coordination are not fully mature [43], and balance is a key component of optimal performance and athletic injury prevention [16, 60], Behm et al. [61] recommended that balance training should be employed before plyometric training in youth and youth athletes. Bruhn et al. [62] concurred, indicating that balance training could provide a pre-conditioning training benefit to subsequent resistance training. Furthermore, balance training prior to plyometric training could improve landing and take-off mechanics. In fact, improved vertical jump performance after five weeks of balance training was attributed to attenuated postural sway promoting a more optimal vertical angle upon jump take-off in recreationally active women [63]. Balance training can enhance proprioceptive afferent feedback to promote more rapid and higher neuromuscular activation during plyometric exercises in athletic and non-athletic populations [64]. Thus, balance prior to resistance training may establish better balance, which permits greater force, torque, and power outputs to enhance performance. In contrast to metastable resistance training, sequenced balance and resistance training appears to be suitable for the general youth population and for youth athletes.

### Study Limitations

This scoping review article has some methodological limitations that warrant discussion. First and foremost, the overall number of identified studies (*N* = 11) and the number of participating individuals is small (*N* = 202 children and adolescents). As a consequence, we did not aggregate and meta-analyze findings from the eleven included RCTs. Instead, we reported single-study outcomes in Table 2 and identified six knowledge gaps in the literature (Table 4). Findings from this scoping review may provide new research avenues for well-designed future studies enabling meta-analyses on the topic (Table 4). When designing future research according to age, maturity status, sex, and expertise level, researchers have to keep in mind that these moderators interact. Accordingly, it is important to apply comparative studies and contrast one moderator only (e.g., sex) while the other moderators (age, maturity status, expertise level) are kept constant across the experimental groups. Second, despite the reporting of power analyses in most studies, overall low sample sizes were observed that ranged from eight to 21 individuals per experimental group.

Third, the active comparator groups varied greatly across studies and ranged from single-mode resistance training to different sequencing schemes of balance and resistance training to regular sport-specific training and finally physical education. Fourth, balance and muscular fitness outcomes varied across the eleven included RCTs. An often encountered criticism of meta-analyses is that they synthesize rather heterogeneous study outcomes (i.e., mixing apples and oranges). We tried to overcome this limitation by reporting single-study findings in our scoping review instead of computing a meta-analysis.

### Recommendations on How to Best Combine Balance and Resistance Training in Youth and Youth Athletes

A series of articles from the same laboratory found that a combination of plyometric and balance training enhanced sprint and shuttle run performances more frequently than plyometric-only training with children [23, 38]. Second, that balance training four weeks prior to resistance training induced either similar or superior performance enhancements when compared with plyometric training after balance training [23], but that the order of balance and resistance training within a single training session did not significantly performance outcomes [22]. Thus, balance training should be an essential training component for youth that is incorporated with the resistance training exercises or introduced at least a month before resistance and plyometric training within a periodized training program. Balance exercises can include not only body mass type calisthenic exercises (e.g., stork stand eyes open and closed), but can also incorporate elastic and rubber bands to disrupt equilibrium when performing a task (e.g., pulling a band attached around an athlete’s chest when performing a tennis stroke), body mass and resistance exercises with low loads performed on stability devices (e.g., BOSU balls, wobble boards, inflatable discs) as well as many other imaginative general and sport-specific balance activities.

## Conclusions and Future Research Avenues

Based on limited-quality evidence (SORT) and good overall methodological quality (PEDRO), this scoping review indicates that metastable resistance training compared with plyometric training performed on stable surfaces appears not to provide additional balance and muscular fitness training benefits. Periodizing a training mesocycle (e.g., four weeks) of balance prior to plyometric training accentuates balance and muscular fitness training adaptations in youth athletes. However, to optimize jump performance, plyometric training on stable surfaces seems to provide greater training adaptations. More research is needed in the form of comparative studies to elucidate the role of age, maturity status, sex, and expertise level on training-induced adaptations following metastable resistance training or sequenced balance and resistance training in youth and youth athletes.

## Supplementary Information

Below is the link to the electronic supplementary material.

Supplementary file1 (DOCX 12 KB)

## Declarations

### Availability of Data and Material

All data supporting the findings of this review are available in this published article or as a supplement.
