# Supplementary material for: Relevance and Effectiveness of Combined Resistance and Balance Training to Improve Balance and Muscular Fitness in Healthy Youth and Youth Athletes: A Scoping Review
Source: Sports Med. 2022 Nov 15;53(2):349–70. doi: 10.1007/s40279-022-01789-7 (PMC9876852; doi:10.1007/s40279-022-01789-7)
Supplement: Supplementary file 1 — Supplementary file1 (DOCX 12 KB) [file 40279_2022_1789_MOESM1_ESM.docx]

**Supplementary material**

**Supplement 1:** Search syntax for the three electronic search engines PubMed, Web of Science, and SPORTDiscus.

PubMed

((“resistance training” [title/abstract] OR “weight-bearing” [Mesh] OR “strength training” [title/abstract] OR “high intensity strength training” [title/abstract] OR “instability resistance training” [title/abstract] OR “instability strength training” [title/abstract] OR “metastable resistance training” [title/abstract] OR “metastable strength training” [title/abstract] OR “plyometric training” [title/abstract] OR plyometric* [title/abstract] OR “plyometric exercise*” [title/abstract] OR “instability plyometric training” [title/abstract] OR “instability with plyometric training” [title/abstract] OR “olympic weightlifting” [title/abstract] OR “complex training” [title/abstract]) AND (“balance training” [title/abstract] OR “balance exercise*” [title/abstract] OR “sensorimotor training” [title/abstract] OR “proprioceptive training” [title/abstract] OR “proprioceptive exercise*” [title/abstract] OR “balance and plyometric training” [title/abstract] OR “perturbation training” [title/abstract]) AND (youth [title/abstract] OR child* [title/abstract] OR adolescent [Mesh] OR young [title/abstract]))

Web of Science

((“resistance training” OR “weight-bearing” OR “strength training” OR “high intensity strength training” OR “instability resistance training” OR “instability strength training” OR “metastable resistance training” OR “metastable strength training” OR “plyometric training” OR plyometric* OR “plyometric exercise*” OR “instability plyometric training” OR “instability with plyometric training” OR “olympic weightlifting” OR “complex training”) AND (“balance training” OR “balance exercise*” OR “sensorimotor training” OR “proprioceptive training” OR “proprioceptive exercise*” OR “balance and plyometric training” OR “perturbation training”) AND (youth OR child* OR adolescent OR young))

SPORTDiscus

((“resistance training” OR “weight-bearing” OR “strength training” OR “high intensity strength training” OR “instability resistance training” OR “instability strength training” OR “metastable resistance training” OR “metastable strength training” OR “plyometric training” OR plyometric* OR “plyometric exercise*” OR “instability plyometric training” OR “instability with plyometric training” OR “olympic weightlifting” OR “complex training”) AND (“balance training” OR “balance exercise*” OR “sensorimotor training” OR “proprioceptive training” OR “proprioceptive exercise*” OR “balance and plyometric training” OR “perturbation training”) AND (youth OR child* OR adolescent OR young))
